# Full-field interferometric imaging of propagating action potentials

**DOI:** 10.1038/s41377-018-0107-9

**Published:** 2018-12-12

**Authors:** Tong Ling, Kevin C. Boyle, Georges Goetz, Peng Zhou, Yi Quan, Felix S. Alfonso, Tiffany W. Huang, Daniel Palanker

**Affiliations:** 1 Hansen Experimental Physics Laboratory, Stanford University, Stanford, CA 94305 USA; 2 Department of Ophthalmology, Stanford University, Stanford, CA 94305 USA; 3 Department of Electrical Engineering, Stanford University, Stanford, CA 94305 USA; 4 Department of Molecular and Cellular Physiology, Howard Hughes Medical Institute, Stanford University, Stanford, CA 94305 USA; 5 Department of Chemistry, Stanford University, Stanford, CA 94305 USA

## Abstract

Currently, cellular action potentials are detected using either electrical recordings or exogenous fluorescent probes that sense the calcium concentration or transmembrane voltage. Ca imaging has a low temporal resolution, while voltage indicators are vulnerable to phototoxicity, photobleaching, and heating. Here, we report full-field interferometric imaging of individual action potentials by detecting movement across the entire cell membrane. Using spike-triggered averaging of movies synchronized with electrical recordings, we demonstrate deformations up to 3 nm (0.9 mrad) during the action potential in spiking HEK-293 cells, with a rise time of 4 ms. The time course of the optically recorded spikes matches the electrical waveforms. Since the shot noise limit of the camera (~2 mrad/pix) precludes detection of the action potential in a single frame, for all-optical spike detection, images are acquired at 50 kHz, and 50 frames are binned into 1 ms steps to achieve a sensitivity of 0.3 mrad in a single pixel. Using a self-reinforcing sensitivity enhancement algorithm based on iteratively expanding the region of interest for spatial averaging, individual spikes can be detected by matching the previously extracted template of the action potential with the optical recording. This allows all-optical full-field imaging of the propagating action potentials without exogeneous labels or electrodes.

## Rapid changes in cell shape reveal its electrical activity

A promising non-invasive technique for measuring electrical activity in neurons and other cells works by observing how cells deform in response to changes in their electric potential. Existing methods for analyzing cells’ electrical activity are invasive and may affect natural cell behavior. Now, Tong Ling and co-workers from Daniel Palanker’s lab at Stanford University in the US demonstrated a method that monitors changes in cell shapes during electrical spikes – or ‘action potentials’ - using quantitative phase microscopy. Action potential occurs when the negatively-charged cell interior depolarizes due to a stimulus, triggering a rapid voltage change across the cell membrane. Ling’s team demonstrated that cells deform in response to these relatively large (0.1 V) voltage shifts, revealing exactly when action potentials occur. Their technique may facilitate non-invasive analysis of neuronal networks and other cellular activities.

## Introduction

Modern methods for detecting electrical activity in cells rely on either electrical or optical recordings, both of which are invasive. Electrical methods require electrodes to be placed adjacent to the cells of interest^1–5^. Optical measurements rely on exogenous fluorescent probes, such as calcium indicators^6^ or transmembrane voltage sensors^7–9^. Ca imaging provides rather low temporal resolution^10^, while fluorescent voltage indicators can be phototoxic and are limited by photobleaching^11^ and heating of the target^9^.

The large changes in transmembrane voltage that take place during action potentials have long been hypothesized to induce changes in the shape of biological cells, which is primarily determined by the balance of intracellular hydrostatic pressure, membrane tension, and strain exerted by the cytoskeleton^12–14^. A layer of mobile ions along the cell membrane exerts additional tension on the lipid bilayer due to their lateral repulsion^15–17^, which makes the membrane tension dependent on the voltage (see Supplementary Fig. S1). A 100 mV depolarization during an action potential increases the tension by ~10 μN m^−1^ (ref. ^15^), which increases the force exerted on the membrane of a 10 μm cell by 0.3 nN. This is expected to deform the cell by decreasing its surface area while preserving volume, thereby making it more spherical. The movement of the cell membrane, called electromotility^15,18^, is expected to follow the voltage change nearly instantaneously, since the magnitude of this force is very significant at the cellular scale: if it were not counteracted by the cytoskeleton, such a force would accelerate the cell by ~600 m/s^2^—much more than what is observed for a 1 nm membrane displacement over 1 ms in an action potential (10^−3^ m/s^2^).

Movements of the cell membrane that accompany action potentials have been detected in the giant squid axon and crustaceans (0.3–5 nm) using the single-point reflection of a laser beam^19–22^, in a crab nerve (5–10 nm) by the shift of a light-obstructing target^23^, and by atomic force microscopy^24^. In mammalian cells, membrane electromotility in HEK-293 and PC-12 cells was measured with atomic force microscopy and piezo sensors (1 nm displacement per 100 mV)^15,25^. The average displacement of the cell membrane was also detected using quantitative phase microscopy (QPM)^26–28^ in HEK-293 cells, whose potential was periodically modulated by a voltage clamp^29^. A recent publication on optical thickness fluctuations in a neuronal cell culture measured with low coherence interferometry^30^ reported changes in the optical path difference (OPD) on the order of ~2 nm. Since this measurement was performed in a transmission geometry, the actual cell membrane displacement was larger than the OPD by the difference in refractive indices of the cell and the medium. With Δ*n*~0.035 (refs. ^31–33^), the membrane displacement should be ~57 nm. This is nearly two orders of magnitude larger than the previous experimental results with neurons in culture^25,34^. In addition, that study did not provide sufficient temporal resolution to assess whether these spikes were actually action potentials, nor did they validate correlations of these fluctuations with the action potentials using electrical recordings. Another recent publication described membrane displacements ranging from 0.2 to 0.4 nm during an action potential measured by averaging the changes in light intensity at the cell edge using a bright-field microscope^34^. However, possible fluid exchange between the cytoplasm and the patch clamp pipette may affect the extent of cellular movements in such experiments.

In this article, we demonstrate the dynamics of cellular movements during action potentials propagating in a cell culture, which are validated by electrical recordings, without affecting the cellular processes. This technique, based on ultrafast QPM with a self-reinforcing sensitivity enhancement, enables a label-free noninvasive optical approach to detect individual action potentials. First, using simultaneous optical and electrical recordings by QPM and a multielectrode array (MEA), we extract a spike-triggered average (STA) template of the optical phase changes in spiking HEK-293 cells during the action potentials. Individual spikes can then be detected optically by matching this template with the phase images without the use of electrical recordings. The detection sensitivity is further enhanced by frame binning and iterative spatial averaging of the expanding region of interest using a self-reinforcing lock-in algorithm.

## Results

### Dynamics of cellular deformation during action potentials

**Fig. 1:**
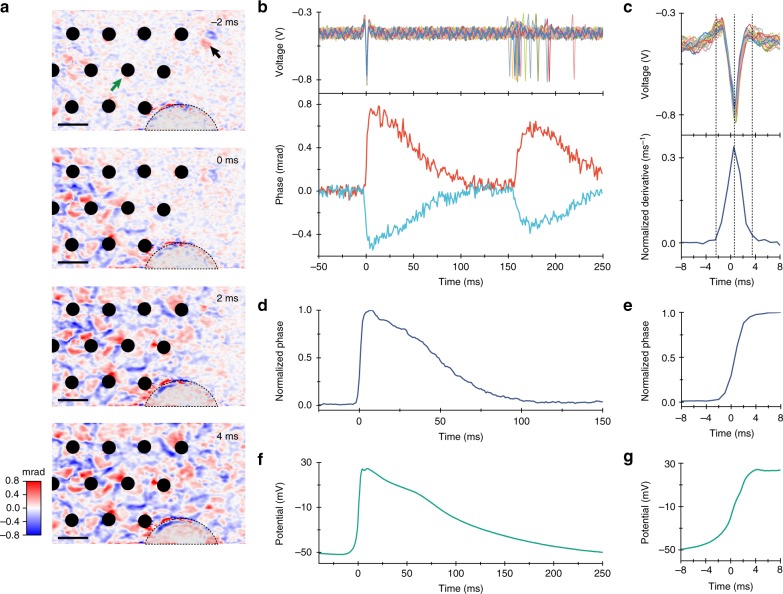
Spike-triggered average of the optical phase shift and the electrical signal during an action potential, obtained by averaging 5130 events. **a** Propagation of the action potential across the field of view over 6 ms (see Supplementary Video 1). The timing of the frames is shown relative to the electrical spike detected on the electrode indicated by a green arrow. Phase changes can be both positive and negative (shown in false color). The black arrow points to a floating cell, which produced a larger phase shift than the action potential, while the dashed line outlines a semicircular section with detached cells. Scale bar: 25 μm. **b** Top row: electrical signals recorded on the reference electrode and aligned to the time of maximum deflection. Subsequent spikes exhibit some degree of natural jitter. Bottom row: average optical phase signals extracted from two individual pixels near the reference electrode at the time of an electrical action potential. **c** Comparison between the electrical signal on the reference electrode (top row) and the time derivative of the optical signal (bottom row). Normalized optical phase signal spatially averaged across the whole FOV (**d**) and its rising edge (**e**), with the spike timing corrected by local delays relative to the spike on the reference electrode. Patch clamp recording of the membrane potential (**f**) and its rising edge (**g**)

To image cellular deformations during the action potentials, we cultured genetically modified HEK-293 cells expressing a voltage-gated sodium channel (Na_V_1.3) and a potassium channel (K_ir_2.1)^35,36^. These cells spontaneously spike in synchrony when cultured confluently^35^. For the simultaneous electrical and optical recordings, cells were plated on a transparent MEA at 90% confluence (see Materials and methods). The sample was illuminated by a superluminescent diode (SLD) with an irradiance of 1 mW/mm^2^. The camera frame rate was 1 kHz, with an exposure duration of 45 μs, and the field of view (FOV) on the sample was 159 × 99 μm^2^. The spontaneous spiking rate of the HEK cells was ~5 Hz, and the recordings were conducted over 17 min to acquire ~5000 spikes. Phase images were retrieved from QPM interferograms using Fourier-domain processing (see Materials and methods). The average optical phase movie recorded during the 50 ms preceding a spike and up to 250 ms after it, which we call the STA optical phase recording, was created by averaging movies from 5130 events (Fig. 1, Supplementary Video 1) aligned in time with the electrical recordings of the action potentials from the MEA. This movie demonstrates a rapid (~4 ms) rise of the phase in the cells during the action potential, followed by a gradual (~100 ms) decline back to the baseline. Extension of the averaging time window beyond the interspike duration to include the following spike demonstrates that natural jitter between the spontaneous spikes results in a smearing of the average. As illustrated in Fig. 1, some pixels exhibit a positive change in phase, while some exhibit a negative change, corresponding to an increase and decrease in the cell thickness, respectively. The action potential wavefront propagated across the FOV in ~6 ms (Fig. 1a), corresponding to a 27 mm/s velocity. In some cells, the optical phase increased on one side and decreased on the other, while in others, it increased at the center and decreased along the boundaries (see Supplementary Fig. S2). The maximum amplitude of the positive phase shift was Δ*ϕ* = 0.86 mrad, corresponding to a Δ*h*~3.2 nm increase in cell thickness $\left( {{\mathrm{\Delta }}\phi = 2\pi /\lambda \cdot {\mathrm{\Delta }}n \cdot {\mathrm{\Delta }}h} \right)$, assuming a refractive index difference of Δ*n* = 0.035 between the cytoplasm (*n*~1.37, refs. ^31,32^) and the cell culture medium (*n* = 1.335 for Tyrode’s solution^33^). This value of Δ*n* also matches the 3.5 rad phase shift in the ~13 μm-thick spiking HEK-293 cell shown in the Supplementary Fig. S2.

By adjusting the timing of the video frames to the local timing of the action potential recorded by the MEA, the phase changes in all pixels can be averaged together for a further improvement in SNR. The average optical waveform recorded during an action potential, shown in Fig. 1d, has a peak SNR of 47.7 dB (calculated with a factor of 20 since the phase signal is related to amplitude rather than power; the peak signal amplitude is 243 times greater than the RMS amplitude of the noise) and is very similar to the shape of the membrane potential recorded with a whole-cell patch clamp (Fig. 1f, g). Note that the tail of the action potential in the patch clamp recording is slightly longer, likely due to differences in the temperature and confluence of the cell culture in the preparation^35^ (see Materials and methods). The general similarity of these waveforms indicates that the cellular deformation during the action potential reflects the changes in transmembrane voltage. In extracellular recordings, the cells and electrodes are capacitively coupled; therefore, the electrical signals correspond to the negative derivative of the intracellular voltage, bandpass filtered over the 43–2000 Hz range. As shown in Fig. 1c, the time derivative of the rising edge of the optically recorded action potential (bottom frame) matches the timing and duration of the electrical spike (top frame), while the derivative of the slow falling edge of the action potential is filtered out by the MEA.

### Noise reduction and template matching for single-spike detection

To reduce the noise for single-spike detection, global fluctuations in the phase image, which originate from the mechanical vibrations of the optical components and 1/*f* noise of the light source, were removed by subtracting the mean value from every frame of the phase image (see Materials and methods). This reduced variations to the shot noise level, set by the well capacity of the camera pixels^37^, with the temporal standard deviation of the phase in a single pixel decreasing from ~3.8 to ~1.9 mrad (see Supplementary Fig. S3). However, this value remains much larger than the maximum phase change during an action potential (0.86 mrad), and additional improvements are needed to achieve single-spike detection.

**Fig. 2:**
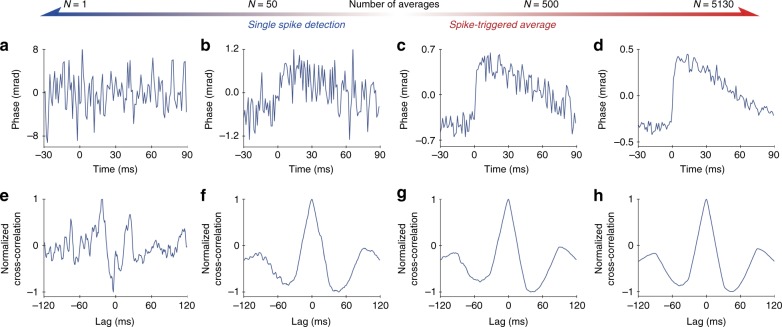
Phase changes in a single pixel as a function of the number of averaged spikes and the corresponding cross-correlation with a phase template of the action potential. Top row (**a**–**d**): when the number of averaged spikes increases from *N* = 1 (**a**) to *N* = 5130 (**d**), the SNR of the phase change increases approximately as $\sqrt N$. Bottom row (**e**–**h**): cross-correlation of the phase trace with the spike template shown in Fig. 1d illustrates that a spike can be detected from 50 averages but not from a single trace. Additional averaging marginally increases the SNR of the cross-correlation. In the left two columns, averages can still be performed by frame binning using an ultrafast camera for single spike detection, while the right two columns illustrate high-fidelity detection of the cellular movement based on a larger number of averages using STA

Using frame binning at a higher imaging rate can increase the SNR of the recording, provided there is sufficient illumination intensity for shorter exposures. In the shot-noise limited regime, when readout noise is negligible, binning *N* frames into one reduces the phase noise by approximately $\sqrt N$. With *N* = 50 frames averaged, the noise in a single pixel decreases to ~0.3 mrad, as shown in Supplementary Fig. S3 and Fig. 2. Further improvement in spike detection was achieved using template matching. When implemented as a matched filter, the output SNR can be increased to *E*/2*N*_0_, where *E* is the total signal energy of the template and *N*_0_ is the power spectral density (W/Hz) of the noise in the original signal before matched filtering^38^. The effect of temporal averaging by the summation of separate spikes synchronized via electrical recordings is illustrated in Fig. 2a–d. Fig. 2b, f shows how an average of *N* = 50 separate spikes, matched to the template shown in Fig. 1d, clearly identifies a spike with no lag (0 ms). In contrast, template matching with no temporal averaging is insufficient (Fig. 2e). The averaging of a larger numbers of spikes, shown in Fig. 2c, d, helps to further increase the SNR, but it does not significantly improve the template matching precision (Fig. 2g, h).

### All-optical spike detection by self-reinforcing sensitivity enhancement

**Fig. 3:**
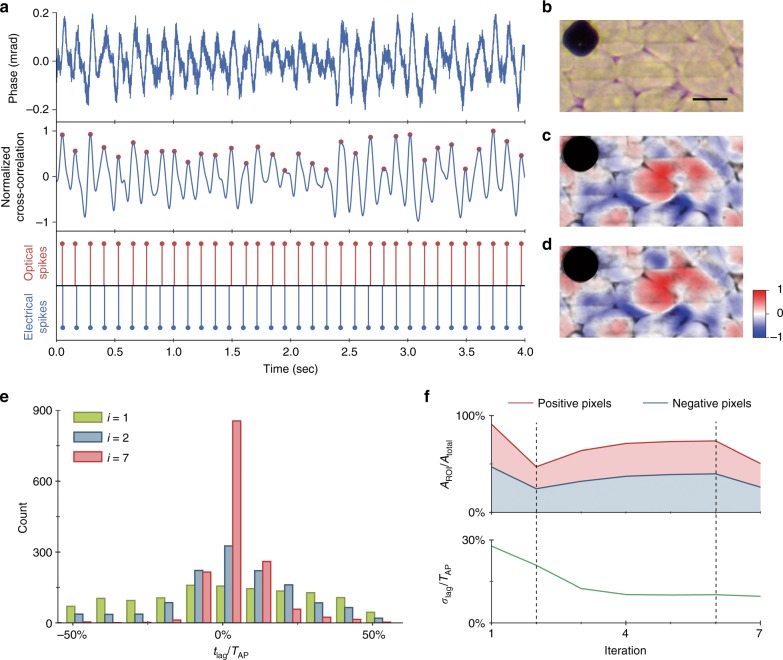
All-optical detection of a single action potential by interferometric imaging using a self-reinforcing lock-in algorithm. **a** Spatially averaged phase signals in the final iteration of the lock-in algorithm, showing a periodic phase signal (top row). Template matching is implemented by cross-correlating this phase signal with an action potential template obtained in a separate recording, and peaks are identified as spikes (middle row). The timing of the optically and electrically detected spikes is compared in the bottom row. A total of 1584 spikes were recorded electrically in this FOV. **b** Spiking HEK-293 cells grown on the MEA, as seen in a bright-field microscope. The (**c**) electrically and (**d**) optically synchronized spike-triggered average movies cross-correlated with the action potential phase template demonstrate nearly identical spike correlation maps during the action potential. **e** Histograms of the time lag between the optically and electrically detected spikes after 1, 2, and 7 iterations show convergence to a distribution centered around zero (mean 3.6%, and standard deviation 9.7% of *T*_AP_). The analysis is based on 1584 spikes recorded electrically in this FOV. **f** Convergence analysis. The dashed lines indicate the successive three stages of the algorithm. During the first iteration, an ROI is selected randomly. In the second stage, from the second iteration to steady-state (sixth iteration here), the ROIs are refined and extended to cover > 70% of the FOV. In the final iteration (seventh iteration here), the ROI is further recharted based on SNR optimization for spatial averaging. The area chart (top row) shows the evolution of the ratio of the ROI area to the total area. The bottom row shows the standard deviation of the time lags between the optically and electrically detected spikes, which converges to 9.7% of the action potential period (*T*_AP_∼120 ms)

To demonstrate all-optical spike detection with QPM, we recorded spontaneously spiking HEK-293 cells at 50 kfps within a smaller FOV of 53 × 26.5 μm^2^ and obtained ground truth electrical spiking data from the MEA for comparison. To achieve sufficiently short exposures, we used a supercontinuum laser providing an irradiance of 4.7 mW/mm^2^ (797–841 nm wavelength), which was sufficiently bright for 10 μs exposures, thus enabling a camera frame rate up to 100 kHz. For all-optical spike detection, i.e., imaging of the action potentials without electrical recordings, we developed an iterative lock-in algorithm that matched the phase shift signals to a spike template that was previously recorded in a different set of HEK cells with the aid of the MEA (Fig. 1d). Using only this template, the algorithm iteratively estimates (a) the timing of the action potentials for temporal averaging and (b) regions of interest (ROI) that spike with the same polarity for spatial averaging (see Materials and methods). After four iterations, the spike timing and spatial distribution of the phase shift polarity stabilize, with electrically and optically triggered average STA maps becoming very similar (Fig. 3c, d and Supplementary Video 2). The time course of the phase changes averaged over the FOV, shown in the top row of Fig. 3a, exhibit an SNR of ~20 dB. After cross-correlation with the spike template (middle row in Fig. 3a), the peaks corresponding to the spike timing can be clearly identified (red dots). The timing of these peaks closely matches the spike timing identified in the electrical recording, as shown in the bottom row of Fig. 3a. The quality of the spike detection is summarized by the standard deviation of the time lags between the optically and electrically detected spikes (Fig. 3e). This distribution starts out flat, showing nearly random spike detection in the first iteration, and quickly narrows around zero delay (perfect detection) with a standard deviation of ~11.6 ms, corresponding to 9.7% of the action potential period (~120 ms). The optical spikes counted in this distribution are only those that were uniquely associated with an electrical spike within one action potential period. Other rarer occurrences, where two or more optical spikes appeared within one electrical period, were considered false positives and occurred at a false discovery rate of 0.07%. The rate of false negative events (the absence of any optical spike during an electrical spike) was 8.5%. In addition to all-optical spike detection in the whole FOV, single spikes of individual cells can also be obtained within the cell boundaries segmented by the bright-field microscope image.

## Discussion

The mechanisms behind the optical phase change during an action potential have been actively debated in the literature. One proposed explanation was a change in the refractive index associated with ions flowing into the cell during the action potential or cell swelling due to water molecules accompanying those ions^24,29^. The number of Na^+^ ions entering the cell during the depolarization phase of an action potential is *N* = *C*_m_*A*Δ*V*_m_/*e*, where Δ*V*_m_ ∼ 100 mV is the transmembrane potential rise, *C*_m_ is the specific membrane capacitance (~0.5  μF cm^−2^, ref. ^15^), *A* is the cell membrane surface area, and *e* is the elementary charge. For a cell 10 μm in diameter, *N* is ∼10^6^, which is <0.03% of the number of Na^+^ ions in a mammalian cell of this size, given that the intracellular Na^+^ ion concentration is ~12 mM (ref. ^39^). Since the change in refractive index is proportional to the variation in ion concentration^40^ and Na^+^ represents <10% of the total amount of ions in a cell, the associated phase change for a cell with an optical thickness of ~3 rad (Supplementary Fig. S2) is not expected to exceed 0.1 mrad—about an order of magnitude below the changes we observed during an action potential. Furthermore, since four water molecules comprise the hydration coat of a sodium ion^41^, a total of ~4 × 10^6^ water molecules enter the cell with the Na^+^ ions during the action potential. They increase the cell volume by approximately a factor of 2.3 × 10^−7^ or its diameter by ~0.7 × 10^−3^ nm, which is three orders of magnitude less than what we observed during the action potential. It follows that neither the change in refractive index due to the influx of ions or the cell swelling from water accompanying those ions can account for the observed phase changes.

Another proposal was that cell swelling might be caused by water diffusion associated with osmotic changes during the action potential. However, this process is relatively slow: mouse cortical neurons swell in a hypotonic solution (144 mOsm/kg H_2_O, compared to 229 mOsm/kg H_2_O in the standard perfusion medium) with a time constant of ~30 s^42^—much longer than the ms-scale rise time of the action potential. Moreover, dilution of the cellular content by the influx of water would result in a decrease of the optical path at the center of a spherical cell and an increase at the boundaries due to cell expansion^42,43^. Our observations were the opposite: the optical phase typically increased at the center and decreased along the cell boundaries. Therefore, changes in the refractive index due to cell swelling from water diffusion are unlikely to be the mechanism behind the rapid phase changes we observed. Cellular deformation due to an increase in membrane tension during depolarization is a more likely explanation of the observed ms-scale dynamics, including the positive and negative phase changes within individual cells.

In summary, our observations demonstrate that mammalian cells deform during the action potential by ~3 nm and that the dynamics of this deformation match the time course of the changes in the cell potential. Phase changes associated with an action potential, measured in a transmission geometry, are below the shot noise limit in a single frame. However, with sufficient temporal and spatial averaging and prior knowledge about the spike shape, leveraged through template matching in our study, cellular deformations during a single action potential can be detected. In principle, phase changes could be increased using a multipass cavity^44^, although it would require a light source with a sufficiently long coherence length, which is likely to increase the amount of speckle in the interferograms. The SNR could also be improved by reducing the shot noise using brighter illumination in conjunction with a camera having a higher sampling rate and/or a larger well capacity^37^. In a reflection geometry, the SNR should be twice as high as in transmission due to the double pass of the reflected beam. The phase change itself $\left( {{\mathrm{\Delta }}\phi = 4\pi \cdot n \cdot {\mathrm{\Delta }}h/\lambda } \right)$ would be ~76 times larger than that in the transmission geometry $\left( {{\mathrm{\Delta }}\phi = 2\pi \cdot {\mathrm{\Delta }}n \cdot {\mathrm{\Delta }}h/\lambda } \right)$, since it does not include the refractive index difference between the cytoplasm and the medium (Δ*n*~0.035). However, for the same illumination power, the intensity of the light reflected from the cell boundary would be smaller by a factor of Δ*n*^2^ (~0.001), and since the phase noise is inversely proportional to the square root of the number of photons detected^28,45^, the 1/Δ*n* improvement in the phase signal would be counteracted by an equivalent increase in the noise. On the other hand, if the full well capacity of the photodetector is the limiting factor rather than the laser power or the cellular damage threshold, an overall gain of 1/Δ*n* in the SNR could be achieved by increasing the illumination intensity.

In conclusion, high-speed QPM can achieve all-optical, label-free, full-field imaging of electrical activity in mammalian cells and may enable noninvasive optophysiological studies of neural networks.

## Materials and methods

### Simultaneous optical and electrical recording by QPM and MEA

**Fig. 4:**
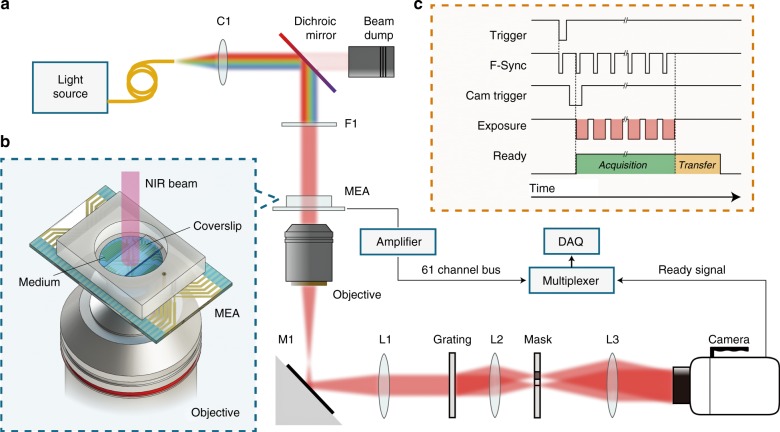
System layout. **a** Ultrafast QPM synchronized with the MEA recording system. Light from a supercontinuum laser is collimated (C1) and filtered by a dichroic mirror and a bandpass filter (F1). An optical phase image of the sample is obtained from the off-axis interferogram captured by the high-speed camera. **b** A transparent MEA plated with spiking HEK cells allows simultaneous near-infrared (NIR) optical recording and extracellular electrical recording. **c** Electrical and optical measurements are synchronized by recording the camera “ready” signal on one of the MEA channels. Trigger signals from the MEA and an external clock control the timing of the captured frames (see Materials and methods)

The setup for quantitative phase imaging was adapted from diffraction phase microscopy^27,28^ and is shown in Fig. 4. Recordings were initially performed at a 1 kHz frame rate using a fiber-coupled SLD (SLD830S-A20, Thorlabs, NJ) for illumination. For faster imaging (50 kHz), a supercontinuum laser (Fianium SC-400-4, NKT Photonics, Birkerød, Denmark) was used. In both cases, light from the fiber was collimated (SLD: F220FC-780, Thorlabs, NJ; the supercontinuum laser had a built-in collimator), and the spectral components of interest were reflected towards the sample arm with a dichroic mirror (FF980-Di01-t1-25×36, Semrock, Rochester, NY). The wavelength range was further restricted to 797–841 nm by an optical bandpass filter (FF01-819/44-25, Semrock, Rochester, NY). Images formed by a 10× objective (CFI Plan Fluor 10×, NA 0.3, WD 16.0 mm, Nikon, Tokyo, Japan) and a 200 mm tube lens (Nikon, Tokyo, Japan) were projected onto a transmission grating (46-074, 110 grooves/mm, Edmund Optics, Barrington, NJ). The first diffraction order passed through unobstructed, while the 0^th^ order was filtered with a 150 μm pinhole mask placed in the Fourier plane of a 4-f optical system, consisting of a 50 mm lens (AF Nikkor 50 mm f/1.8D, Nikon, Tokyo, Japan) and a 250 mm biconvex lens (LB1889-B, Thorlabs, NJ). Interferograms were formed on the camera sensor (Phantom v641, Vision Research, Wayne, NJ), which has a full well capacity of 11,000 electrons (digitized to 12 bit). At up to 1000 fps, the camera can operate at a field size of up to 2560 × 1600 pixels, while at 50,000 fps, the FOV is reduced to 256 × 128 pixels. To decrease the memory storage requirements, we used a FOV of 768 × 480 pixels at 1000 fps. The external clock signal (Model 2100 Isolated Pulse Stimulator, A-M Systems, Sequim, WA) provided to the camera’s F-Sync input was triggered by the falling edge of a TTL trigger generated by the MEA. This trigger was also delivered to the camera via a digital delay generator (DG535, Stanford Research Systems, Sunnyvale, CA) to start the image acquisition in synchrony with the MEA recordings. The signal from the camera indicating that it is ready for a trigger, which was high during both image acquisition (10 s for 768 × 480 pixels at 1000 fps) and data transfer to the nonvolatile memory of the camera (~8 s for 768 × 480 pixels at 1000 fps), was recorded by the data acquisition card (DAQ) of the MEA system to mark the start of each movie.

To retrieve the phase image, the Fourier transform of each interferogram was first calculated. The first diffraction order was then centered and isolated with a low-pass Gaussian filter. To monitor the changes in the phase image, the first interferogram in each movie sequence served as a reference. The phase difference between the reference and subsequent interferograms in the sequence was calculated by taking the argument of the pointwise complex division of the inverse Fourier transform of each filtered interferogram in the series and the filtered reference interferogram^46^. Fluctuations resulting from 1/*f* noise of the illumination source were eliminated by subtracting the average of each phase image to achieve zero mean over the FOV. Highly noisy pixels, typically corresponding to a region obstructed by the electrodes of the MEA, were excluded from the analysis. The phase retrieval process was accelerated using a graphics processing unit (Tesla K40c, Nvidia, Santa Clara, CA).

Electrical signals were recorded using a custom 61-channel MEA system built on a transparent substrate with ITO leads^47,48^. The recording electrodes were 10 μm in diameter and laid out in a hexagonal lattice with 30 µm spacing between the neighboring electrodes and 30 µm spacing between the rows. Platinum black was electrodeposited on the electrodes prior to every recording. The signals were amplified with a gain of 840 and filtered with a 43–2000 Hz bandpass filter. Signals were sampled at 20 kHz using a National Instruments DAQ (NI PCI-6110, National Instruments, Austin, TX). The “ready” signal from the high-speed camera (Phantom v641, Vision Research, Wayne, NJ), marking the start of an image sequence acquisition, was used to synchronize the electrical and optical recordings.

### SNR optimization for spatial averaging

Since the pixels display both positive and negative phase shifts during the action potential, proper spatial averaging should take into account the polarity of the phase shift in each area:

1 $$\bar \varphi \left( t \right) = \frac{1}{N}\mathop {\sum }\limits_{ij} \varphi _{ij}(t) \cdot g_{ij}$$

where *N* is the total number of pixels, *φ*_*ij*_(*t*) is the phase shift at time *t* in pixel (*i,j*) and *g*_*ij*_ is the sign of the overall phase shift in that area. Note that this is different from averaging the absolute value of the phase, since *g*_*ij*_ remains constant for each given pixel, while the sign of the noise changes over time. Averaging of the absolute values would not reduce the noise. A subset of the FOV can be selected to optimize the SNR of the spatially averaged phase signal based on the knowledge of the maximum phase change during the action potential (signal amplitude Φ_*ij*_) and noise level (Δ_*ij*_) in each pixel. Both Φ_*ij*_ and Δ_*ij*_ are sorted across all pixels according to decreasing SNR into arrays Φ_*k*_ and Δ_*k*_. Then, the maximum SNR for spatial averaging can be calculated as follows:

2 $$\overline {{\mathrm{SNR}}} = \mathop {{\max }}\limits_M \frac{{\mathop {\sum }\nolimits_{k = 1}^M {\mathrm{\Phi }}_k}}{{\sqrt {\mathop {\sum }\nolimits_{k = 1}^M {\mathrm{\Delta }}_k^2} }},M \in \left[ {1,N} \right] \cap {\Bbb Z}^ + $$

The first *M* pixels of the sorted arrays are then selected as the optimal subset for spatial averaging in that area.

### Self-reinforcing lock-in spike detection

**Fig. 5:**
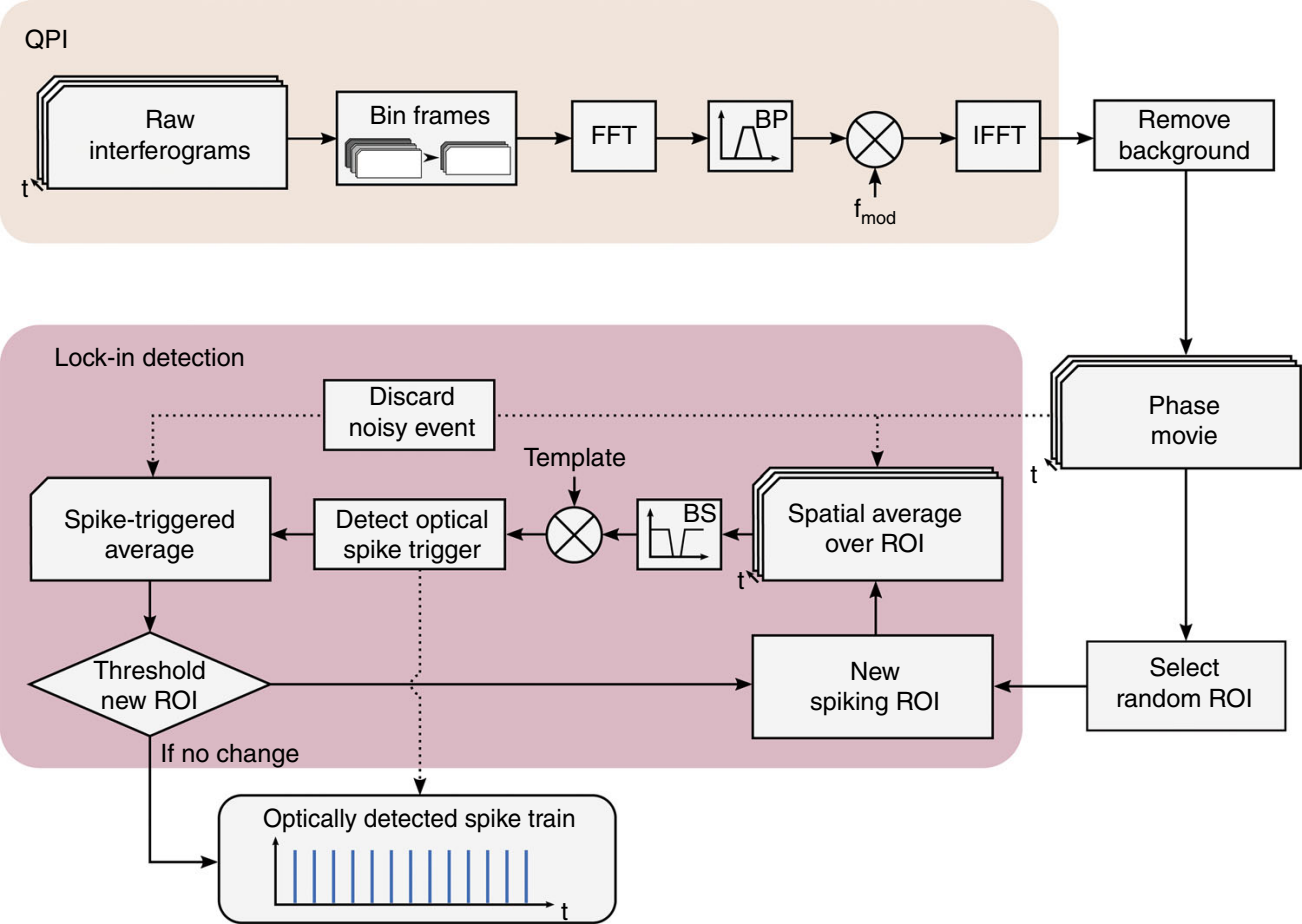
Block diagram of QPM data processing and the self-reinforcing lock-in algorithm for all-optical spike detection. After QPM processing, frame binning, and background removal, a random region of interest (ROI) is selected for the first iteration of the lock-in detection loop. The phase is spatially averaged across the new spiking ROI, band-stop filtered and correlated with the spike template. This correlation output is used to detect an optical spike trigger, which is applied to the original frames of the phase movie to produce a spike triggered average (STA). Noisy frames are detected and discarded at this step. The STA is then used to threshold a new spiking ROI and the loop repeats. With each iteration, the estimate of the ROI and the resulting STA are improved, and the loop exits when the ROI converges to a stable result

The SNR of individual pixels in the phase image is too low to reliably detect an action potential. However, since the spiking in a confluent culture of HEK cells is synchronized, spatial averaging can improve the SNR of the collective measurement. This spatial averaging must be applied taking into account the positive and negative phase shifts across the cell, as described previously. However, since neither the distribution of the phase shift across the FOV nor the spike timing in the optical recordings are known *a priori*, an iterative lock-in spike detection algorithm was developed to detect spikes in the noisy raw recording (see Fig. 5 and Supplementary Fig. S4).

Since the SNR of individual pixels is insufficient for reliably determining the sign of the phase shift at that location, initial spiking ROI are chosen randomly. Phase changes are spatially averaged over the ROIs with a positive and negative sign randomly assigned to each pixel, yielding a single trace showing the displacement of the whole ROI. The randomly spatially averaged phase signal has a slightly improved SNR compared to single pixels. The resulting phase signal is filtered to remove mechanical vibrations and then cross-correlated with a characteristic template of the displacement during the action potential, which was obtained from a separate experiment using the reference MEA electrical recording to perform spatiotemporal spike-triggered averaging. The resulting cross-correlogram gives an estimate of the spike timing in the spatially averaged phase signal, with spike times corresponding to peaks above a set prominence threshold. The detected spikes are used to create a STA from the original movie, corresponding to a single action potential seen across the entire FOV. Each pixel in the STA movie is then correlated with the spike displacement template again to measure the similarity between that location’s displacement and the template, which we summarize as the lock-in image *L* defined as

3 $$L\left( {x,y} \right) = \mathop {\sum }\limits_t \phi (x,y,t) \cdot T(t)$$

Here, *ϕ*(*x*, *y*, *t*) is the phase movie and *T*(*t*) is the displacement template each pixel is expected to follow. The result provides an improved estimate of which parts of the FOV move together, and this ROI is used to start a new iteration of the loop. The process is repeated until the fraction of updated pixels between the new and old ROI decreases below a set threshold (200 pixels), and a final SNR optimization step reduces the size of the ROI. A step-by-step diagram of how the data evolves throughout the lock-in algorithm is shown in Supplementary Fig. S4.

Each iteration of the lock-in detection algorithm improves the estimate of the ROI and thus the quality of the optically detected spike train and the STA movie. Figure 3f shows that convergence occurs in four iterations. The standard deviation of the delay between the detected optical and electrical spikes decreases with subsequent iterations (Fig. 3e), converging into a narrower distribution around zero delay (perfect detection). The timing of the spikes detected in the initial iteration is nearly random and contains a large number of false positives and false negatives, but the final distributions are narrowly confined with an 11.6 ms standard deviation.

### Sample preparation

Spontaneously spiking HEK cells expressing the Na_v_ 1.3 ion channel were originally developed by Adam Cohen’s group at Harvard University^35^. The cells were grown in a 1:1 mixture of Dulbecco’s modified Eagle medium and F-12 supplement (DMEM/F12). The medium contained 10% fetal bovine serum, 1% penicillin (100 U/mL), streptomycin (100 µg/mL), geneticin (500 µg/mL), and puromycin (2 µg/mL). To spontaneously spike, HEK cells need to express not only Na_v_ 1.3 but also the K_ir_ 2.1 ion channel. Hence, they were transfected with the plasmid pIRES-hyg-K_ir_2.1 AMP Resistance using CalFectin as the transfection reagent. Thirty minutes after a medium change, 3 µL of 2.2 µg/µL of the plasmid was mixed with 1 mL DMEM. Then, 3 µL of CalFectin was added to the solution, and 10–15 min later, the mixture was added to the cell culture. Approximately 6 h later, the cell culture was replaced, and the cells were used in experiments 24 h later.

Spiking HEK-293 cells were plated on the MEA coated with poly-d-lysine (P6407, Sigma-Aldrich) at a density of 2000 cells/mm^2^ one day before the recording. Culture medium (DMEM + 10% FBS) filtered with a sterile vacuum filter (SCGP00525, EMD Millipore, Darmstadt, Germany) was used to wash away any floating particles in the MEA chamber, using two applications of 800 μL each time. Two hours prior to the recording, 7/8 of the culture medium was replaced with the recording medium (Tyrode’s solution, in mM: 137 NaCl, 2.7 KCl, 1 MgCl_2_, 1.8 CaCl_2_, 0.2 Na_2_HPO_4_, 12 NaHCO_3_, 5.5 d-glucose). 1/8 of the culture medium was kept to avoid osmotic shock. Extra recording medium was then aspirated until the fluid just filled the 1.5 mm gap between the coverslip and the MEA (see the diagram in Fig. 4b and the actual bright-field image of the confluent HEK cells on MEA in Supplementary Fig. S2). The temperature was maintained at 29 °C during the recordings.

### Whole-cell patch clamp

Spiking HEK-293 cells were placed in the same bath solution as that used for QPI at room temperature (25 °C). Glass micropipettes were pulled from borosilicate glass capillary tubes (Warner Instruments) using a PC-10 pipette puller (Narishige) and were loaded with internal solution containing (in mM) 125 potassium gluconate, 8 NaCl, 0.6 MgCl_2_, 0.1 CaCl_2_, 1 EGTA, 10 HEPES, 4 Mg-ATP, and 0.4 Na-GTP (pH 7.3, adjusted with NaOH; 295 mOsm, adjusted with sucrose). The resistance of the pipettes filled with internal solution varied between 2 and 3 MΩ. After setting up the whole cell configuration, the membrane potential during the spontaneous action potentials of spiking HEK cells was monitored with a Multiclamp 700B amplifier (Molecular Devices) under the current clamp mode.

## Supplementary information

Supplementary Information
Supplementary Video S1
Supplementary Video S2
