# Supplementary material for: Full-field interferometric imaging of propagating action potentials
Source: Light Sci Appl. 2018 Dec 12;7:107. doi: 10.1038/s41377-018-0107-9 (PMC6290013; doi:10.1038/s41377-018-0107-9)
Supplement: Supplementary file 1 — Supplementary Information [file 41377_2018_107_MOESM1_ESM.docx]

Supplementary Information for

**Full-Field Interferometric Imaging of Propagating Action Potentials**

Tong Ling, Kevin C. Boyle, Georges Goetz, Peng Zhou, Yi Quan, Felix S. Alfonso, Tiffany W. Huang and Daniel Palanker

**Corresponding Authors:** Tong Ling, Daniel Palanker.

**E-mail:** [tongling@stanford.edu](mailto:tongling@stanford.edu), [palanker@stanford.edu](mailto:palanker@stanford.edu)

**This PDF file includes:**

Figs. S1 to S4

Captions for Videos S1 to S2

**Other supplementary materials for this manuscript include the following:**

Videos S1 to S2

**Supplementary Figures**


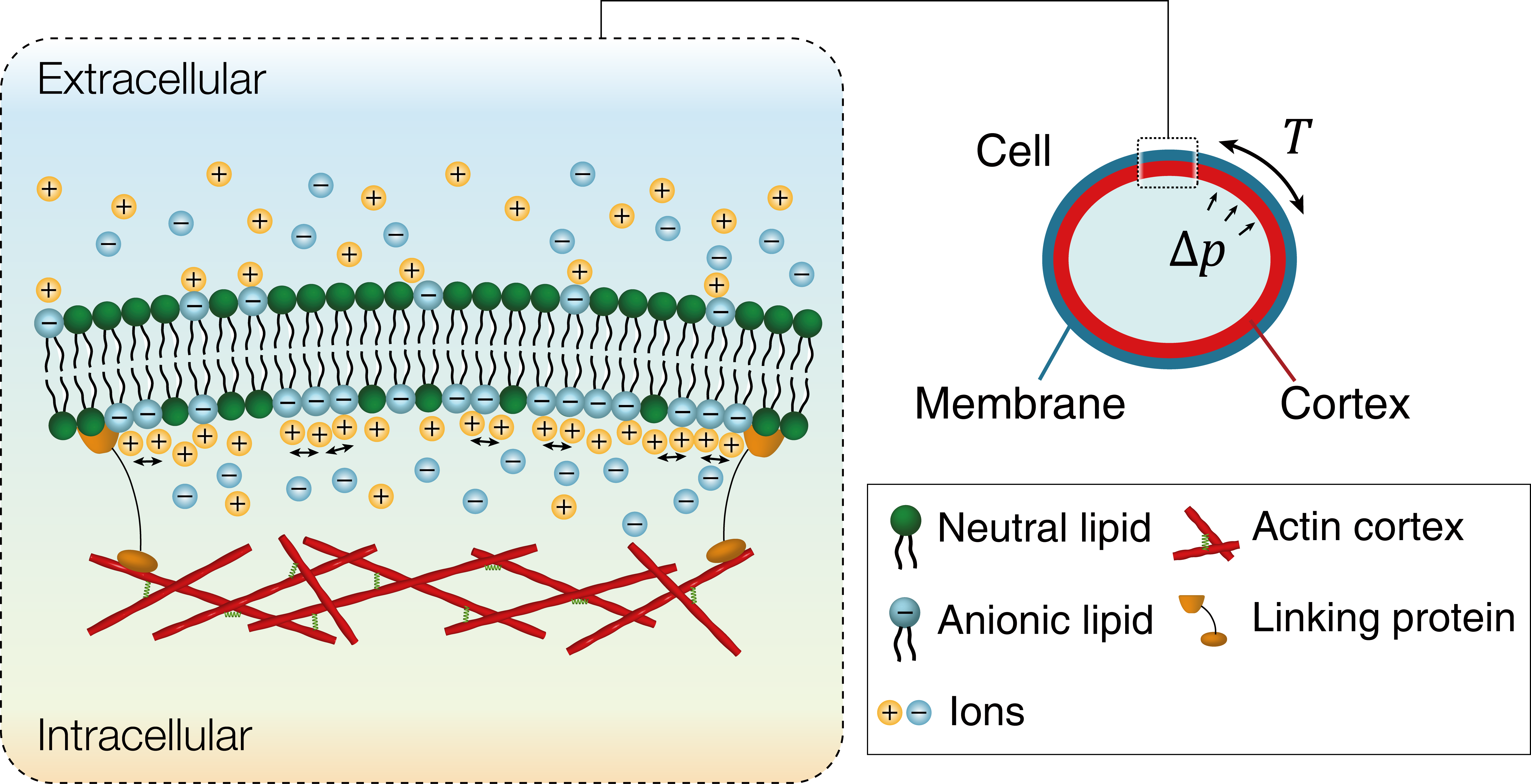


**Figure S1:** The shape of a cell is determined by the balance of hydrostatic pressure ∆*p*, cytoskeleton strain and membrane tension *T*, which depends on concentration of ions along its surface. Upon depolarization, increased surface tension of the lipid bilayer leads to cell deformation.


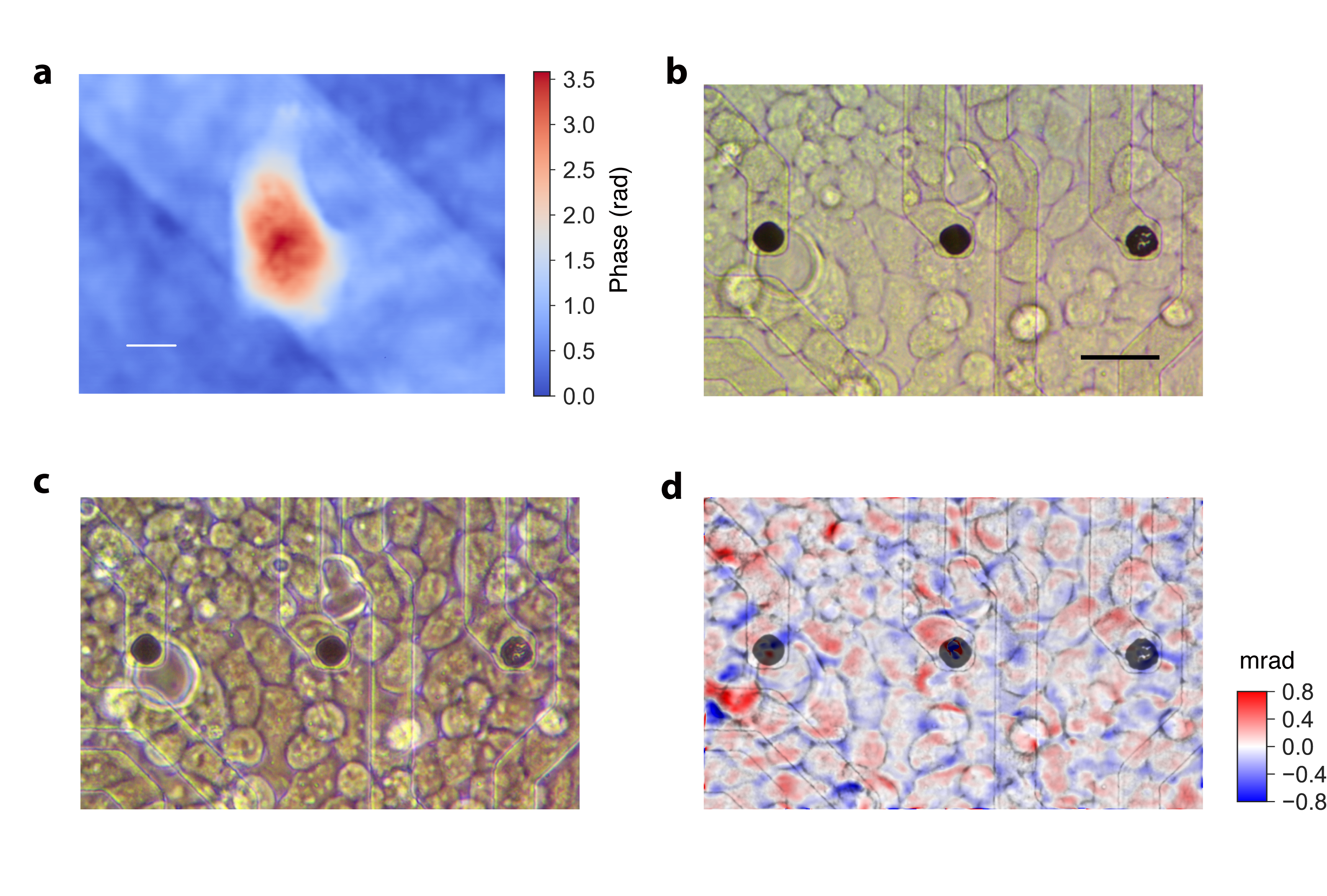


**Figure S2:** Micrographs of spiking HEK-293 cells on MEAs. (a) Phase image of a spiking HEK cell above an ITO wire. Scale bar: 10 μm. (b) Bright-field and (c) phase contrast images of cells plated on 61-electrode MEA with transparent indium tin oxide (ITO) leads. Dark circles are Pt-coated electrodes. Scale bar: 25 μm. (d) Optical phase signal blended with the bright-field image over the same field of view as in (b) and (c).


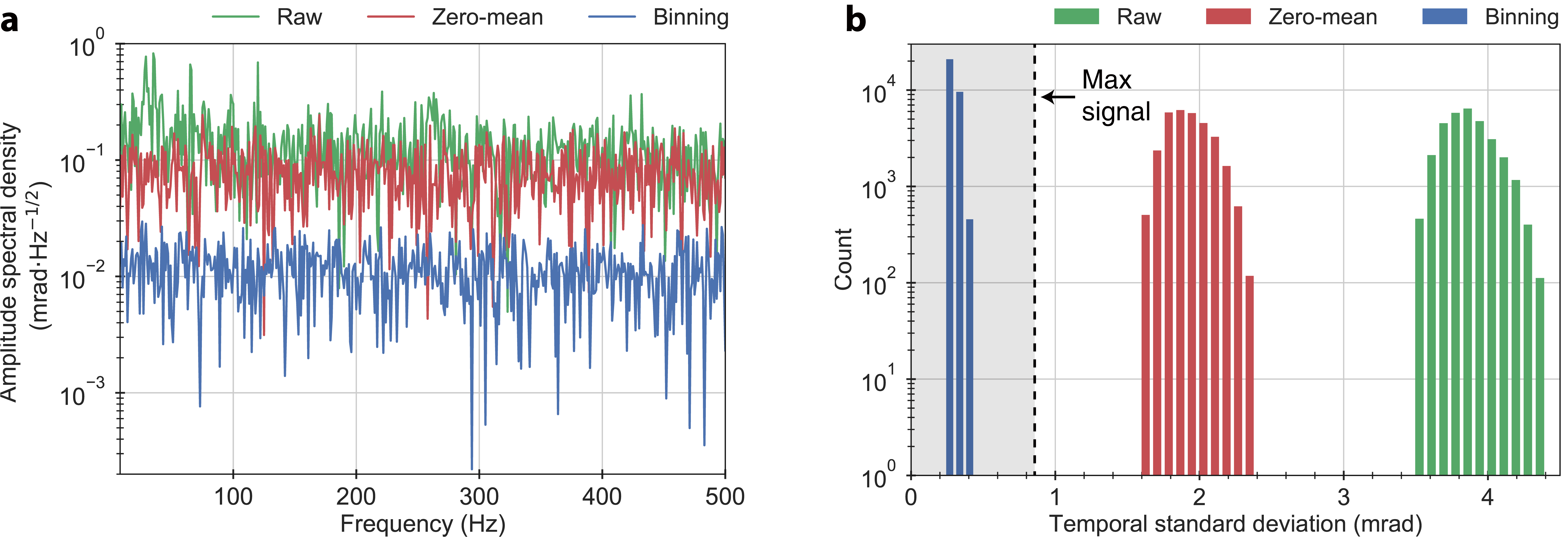


**Figure S3:** Noise reduction for single-spike detection. (a) Spectral density of the phase noise in a single pixel. Raw phase data is shown in green. Background subtraction (red) suppresses the effect of mechanical vibrations and variations in laser intensity, and reduces the phase noise close to the shot-noise limit. Binning 50 frames into one further reduces the noise to ~${10}^{-2} mrad\cdot\mathrm{Hz}^{-1/2}$. (b) Temporal distribution of noise (standard deviation) in all pixels. With background removal and binning of 50 frames (blue), noise drops below the maximum signal level.


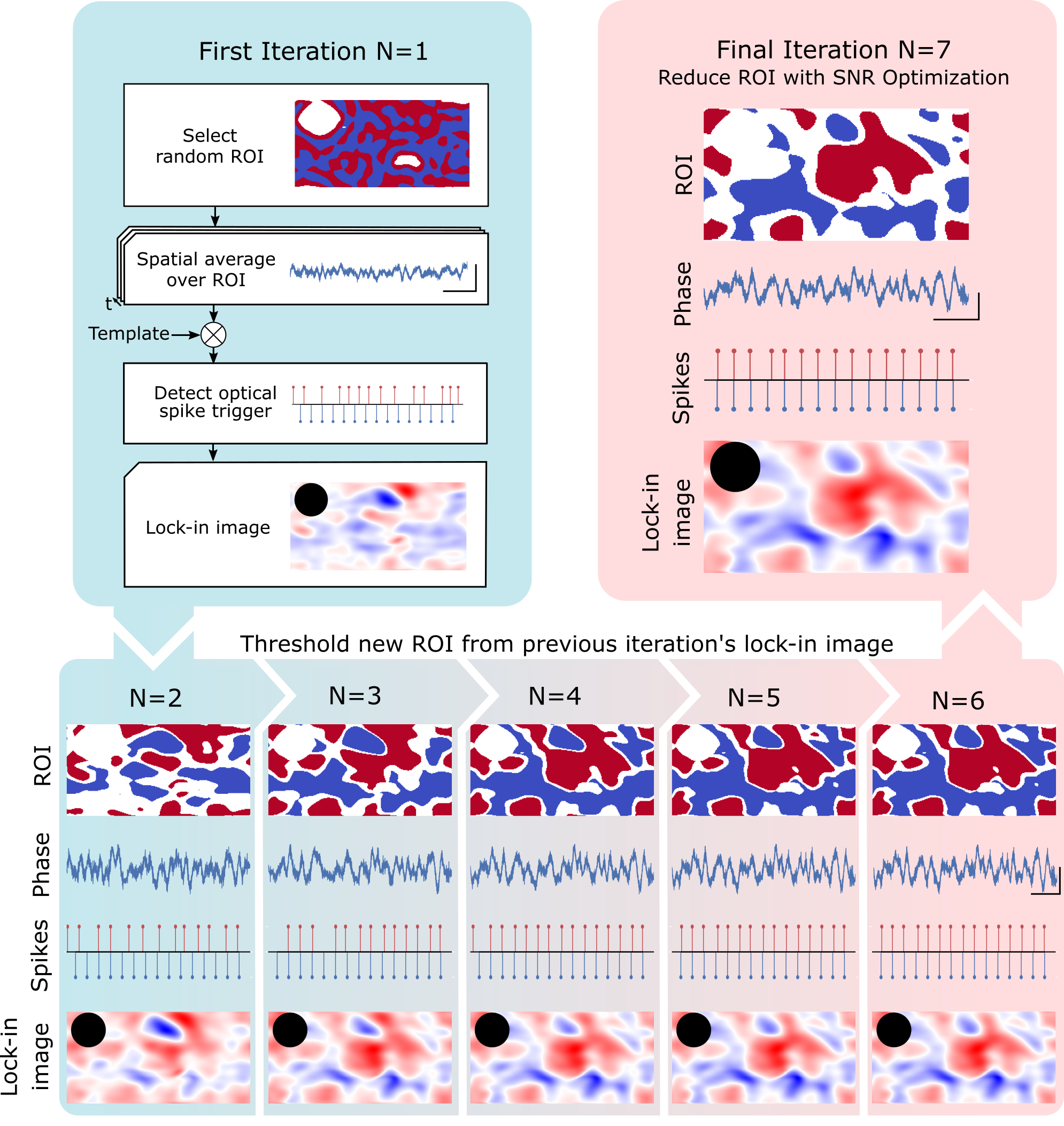


**Figure S4:** Diagram of the self-reinforcing lock-in algorithm for the dataset shown in the article. In the first iteration of the loop, a random ROI is selected, as shown with red regions randomly assigned to positive displacement and blue regions to negative displacement. The phase is spatially averaged across this ROI (initially yielding low signals due to the random averaging), and potential spikes are detected with the spike template. A spike triggered average (STA) is then formed with frames triggered by the detected spike times, and the correlation of each pixel with the template gives the lock-in image as a summary of how well the STA matches the expected deformations. The lock-in image is thresholded to yield an updated estimate for the ROI in the next iteration. Each panel shows the ROI at the corresponding step, the average phase across that ROI, a selection of the (top) detected optical spikes and (bottom) ground truth electrical spikes, and the resulting lock-in image. In the final iteration, the size of the ROI is reduced by an SNR optimization step. Scalebars on the phase traces are all 0.2 mrad in the y-axis and 250 ms in the x-axis. Each set of the phase traces and detected spikes is a 2 second subset of the full dataset used in that iteration.

**Supplementary Videos**

**Video 1:** Spike-triggered average (STA) phase movie of two action potentials propagating across the FOV of 159 × 99 μm^2^.

**Video 2:** Comparison of two STA phase movies based on optically- and electrically- detected spikes.
